# Recombinant Newcastle disease virus rL-RVG enhances the apoptosis and inhibits the migration of A549 lung adenocarcinoma cells via regulating alpha 7 nicotinic acetylcholine receptors in vitro

**DOI:** 10.1186/s12985-017-0852-z

**Published:** 2017-10-03

**Authors:** Yulan Yan, Chunxiang Su, Min Hang, Hua Huang, Yinghai Zhao, Xiaomei Shao, Xuefeng Bu

**Affiliations:** 1 Department of Respiratory Medicine, Affiliated People’s Hospital of Jiangsu University, Zhenjiang, Jiangsu 212002 People’s Republic of China; 2 Department of Internal Medicine, Clinical Medicine College of Jiangsu University, Zhenjiang, Jiangsu 212013 People’s Republic of China; 3 Department of General Surgery, Affiliated People’s Hospital of Jiangsu University, Zhenjiang, Jiangsu 212002 People’s Republic of China

**Keywords:** Recombinant Newcastle disease virus, Rabies virus glycoprotein, Lung adenocarcinoma, α7 nicotinic acetylcholine receptors, Apoptosis

## Abstract

**Background:**

The aim of this study were to investigate the possible pro-apoptotic mechanisms of the recombinant Newcastle disease virus (NDV) strain rL-RVG, which expresses the rabies virus glycoprotein, in A549 lung adenocarcinoma cells via the regulation of alpha 7 nicotinic acetylcholine receptors (α7 nAChRs) and to analyze the relationships between α7 nAChR expression in lung cancer and the clinical pathological features.

**Methods:**

α7 nAChR expression in A549, LΑ795, and small-cell lung carcinoma (SCLC) cells, among others, was detected using reverse transcription polymerase chain reaction (RT-PCR). The optimal α7 nAChR antagonist and agonist concentrations for affecting A549 lung adenocarcinoma cells were detected using MTT assays. The α7 nAChR expression in A549 cells after various treatments was assessed by Western blot, immunofluorescence and RT-PCR analyses. Apoptosis in the various groups was also monitored by Western blot and TUNEL assays, followed by the detection of cell migration via transwell and scratch tests. Furthermore, α7 nAChR expression was examined by immunohistochemistry in lung cancer tissue samples from 130 patients and 40 pericancerous tissue samples, and the apoptotis in lung adenocarcinoma tissue was detected by Tunel assay, Then, the expression levels and clinicopathological characteristics were analyzed.

**Results:**

Of the A549, LΑ795, SCLC and U251 cell lines, the A549 cells exhibited the highest α7 nAChR expression. The cells infected with rL-RVG exhibited high RVG gene and protein expression. The rL-RVG group exhibited weaker α7 nAChR expression compared with the methyllycaconitine citrate hydrate (MLA, an α7 nAChR antagonist) and NDV groups. At the same time, the MLA and rL-RVG treatments significantly inhibited proliferation and migration and promoted apoptosis in the lung cancer cells (*P* < 0.05). The expression of α7 nAChR was upregulated in lung cancer tissue compared with pericancerous tissue (*P* = 0.000) and was significantly related to smoking, clinical tumor-node-metastases stage, and histological differentiation (*P* < 0.05). The AI in lung adenocarcinoma tissue in high-medium differentiation group was lower than that in low differentiation group (*p* < 0.01).

**Conclusions:**

An antagonist of α7 nAChR may be used as a molecular target for lung adenocarcinoma therapy. Recombinant NDV rL-RVG enhances the apoptosis and inhibits the migration of A549 lung adenocarcinoma cells by regulating α7 nAChR signaling pathways.

**Electronic supplementary material:**

The online version of this article (10.1186/s12985-017-0852-z) contains supplementary material, which is available to authorized users.

## Background

Lung cancer is the most common cause of cancer-related mortality in the world [1]. It is a heterogeneous disease with two different pathological types: non-small-cell lung cancer (NSCLC), which accounts for 80% of all lung cancer, and small-cell lung cancer (SCLC). In addition, 30–50% of NSCLC instances are adenocarcinoma. Currently, by the time they are diagnosed with lung cancer, most patients have lost the opportunity for surgery. These patients are only given radiotherapy, chemotherapy or a combination of two or three treatments, and the five-year survival rate of lung cancer patients has remained low. Therefore, novel lung cancer treatments are urgently needed.

Oncolytic therapy, a viral therapy for human cancers, is a novel biological treatment that integrates gene therapy and immunotherapy. Newcastle disease virus (NDV) excels as an oncolytic agent that displays unmatched oncolytic activity among naturally occurring viruse [2]. Multiple studies have shown that oncolytic viruses are well tolerated by cancer patients who benefit from their therapeutic potential via oncolytic virotherapy, especially when virotherapy is implemented in the absence of immunostimulatory interventions [3]. In addition to excelling as an oncolytic agent, NDV also could be a promising vaccine carrier [4–6]. The present study investigates a recombinant avirulent NDV LaSota strain (wild-type NDV strain) expressing the rabies virus glycoprotein (rL-RVG), which is able to suppress lung cancer cell growth and promote lung cancer cell apoptosis to a greater extent than the wild-type NDV strain [7]. rL-RVG is safe in several species and can even be used as a vaccine to induce long-lasting, systemic protective immunity [8].

Neuronal nicotinic acetylcholine receptors (nAChRs) are prototypic, ligand-gated ion channels permeable to either calcium or sodium [9]. There are several types of nAChRs, which comprise either combinations of two different subunit types (α and β) or five copies of the same subunit symmetrically arranged around a central ion pore [10]. Nine types of α subunits (α2–α10) and three types of β subunits (β2–β4) have been cloned and defined. Approximately 80% of lung cancer cases are associated with smoking; therefore, smoking has been recognized as the major cause of lung cancer in developing countries [11]. Nicotine is one of the most prevalent and important components of cigarette smoke and is an agonist of nAChRs. Recent studies have shown that α7 is the main nAChR subunit that triggers the proliferative effects of nicotine in cancer cells [12]. Therefore, α7 nAChR might be a valuable molecular target for the treatment of lung cancer.

Methyllycaconitine citrate hydrate (MLA), a phytotoxin, is a specific competitive antagonist of α7 nAChR and has been extensively used to study the function and distribution of α7 nAChR subtype. Also, MLA has high affinity, high selectivity and functional identification for α7 nAChR, which makes it be the first choice for treatment or drug selection for α7 nAChR. In addition, acetylcholine bromide (ACB) is as an acetylcholine agonist in our research.

A previous study of ours showed that rL-RVG was able to suppress lung cancer cell growth and promote lung cancer cell apoptosis to a greater extent than the wild-type NDV strain [7]. However, the mechanism of how rL-RVG induces pro-apoptosis pathways in lung cancer cells has not yet been elucidated. The 198–214 amino acid sequence of RVG is highly homologous with the 30–56 sequence in λ-bungarotoxin, which is able to bind nAChRs. In addition, α7 nAChRs are ubiquitous in human lung cancer cells [13]. Thus, in this study, we demonstrate that the mechanism underlying rL-RVG-induced anti-cancer activity in A549 lung cancer cells could be that rL-RVG enhances the apoptosis and inhibits the migration of A549 cells via regulating α7 nAChRs; we also present relationships between α7 nAChR expression and the clinical features of this condition.

## Methods

### Materials

The wild-type NDV LaSota strain, the recombinant NDV strain (rL-RVG), and the anti-NDV antibody were kindly provided by the Harbin Veterinary Research Institute (Harbin, China) and were stored at −70 °C. The A549 lung adenocarcinoma cells were purchased from the Cell Culture Center of the Basic Institute of Medical Sciences, Peking Union Medical College (Beijing, China). Mouse lung adenocarcinoma LΑ795, human SCLC and human glial cell tumor U251 cells were all stored in our laboratory and were reserved for the present experiment. 3-[4,5-Dimethyl-2-thiazolyl]-2, 5-diphenyl-2H-tetrazolium bromide (MTT) was obtained from Amresco; all polymerase chain reaction (PCR) primers were purchased from the Shanghai Sangon Biological Engineering Technology & Services Co., Ltd. (Shanghai, China). PCR master mix was obtained from Toyobo (Osaka, Japan), and a reverse transcription kit was purchased from Thermo Fisher Scientific (MA, USA). TRIzol was purchased from Invitrogen Life Technologies. Rabbit polyclonal anti-caspase-3, anti-caspase-8, anti-caspase-9, and anti-Bax and mouse monoclonal anti-Bcl-2 were purchased from Boster (Wuhan, China); rabbit polyclonal anti-α7 nAChR was purchased from Abcam (London, UK). Methyllycaconitine citrate hydrate (MLA) was obtained from Santa Cruz (California, USA); acetylcholine bromide (ACB) was purchased from Sigma-Aldrich (St. Louis, MO, USA). Mouse monoclonal anti-rabies virus was purchased from Santa Cruz (California, USA). Horseradish peroxidase (HRP)-conjugated goat anti-rabbit, HRP-conjugated goat anti-mouse, Cy3-conjugated goat anti-rabbit and the fluorescein isothiocyanate (FITC)-conjugated goat anti-mouse antibodies were purchased from CoWin Biotech, Co., Ltd. (Beijing, China). HRP-conjugated AffiniPure rabbit anti-chicken antibody was obtained from EarthOx Life Sciences (Millbrae, CA, USA), and an anti-chicken antibody was purchased from MedImmune. PVDF membranes (Millipore, CA, USA) and Luminata™ Crescendo Western HRP Substrate were also purchased (Millipore, Middlesex, MA, USA). Dulbecco’s Modified Eagle’s medium (DMEM), fetal bovine serum (FBS), trypsin, and EDTA-2Na were obtained from Gibco (Gibco Life Technologies, Carlsbad, CA, USA). A terminal deoxynucleotidyl transferase-mediated dUTP-biotin nick end-labeling (TUNEL) assay kit was purchased from Nanjing Kaiji Biotechnology Development Co., Ltd., (Nanjing, China). All other supplies used for cell culture were purchased from Costar, Corning (Corning, NY, USA).

### Methods

#### Cell culture

Human lung adenocarcinoma A549, LΑ795, SCLC and U251 cells were maintained in DMEM with 10% FBS at 37 °C with 5% CO_2_ and 100% humidity.

#### Reverse transcription PCR (RT-PCR) analysis

Total RNA was extracted from A549, LΑ795, SCLC, and U251 cells separately using TRIzol. Then, cDNA was synthesized with Oligo (dT) primers (Thermo, MA, USA) and Moloney murine leukemia virus reverse transcriptase (Thermo, MA, USA); 1/10 cDNA was used for PCR amplification. The following PCR protocol was used: initial denaturation at 94 °C for 5 min, followed by 30 cycles at 94 °C for 30 s, annealing at 55 °C (NDV), 53 °C (RVG), 55 °C (α7 nAChR) or 56 °C (GAPDH) for 30 s, and extension at 72 °C for 30 s. The final extension was performed by an incubation step at 72 °C for 10 min. The PCR products were subjected to electrophoresis in 2% agarose gel (Sigma-Aldrich, MO, USA) and visualized with ethidium bromide (Sigma-Aldrich, MO, USA). The bands were analyzed with Quantity One software, version 4.62 (Bio-Rad, CA, USA). All primers used are listed in Additional file 1: Table S1.

#### MTT assays

rL-RVG (109.8 EID50/mL), wild-type NDV (109.8 EID50/mL), MLA and ACB were diluted by 10^3^, 10^4^ and 10^5^ with serum-free DMEM. A549 cells in the logarithmic growth phase were plated at 1 × 10^4^ cells/well in 96-well plates, incubated overnight, and were either treated with diluted rL-RVG, wild-type NDV, MLA, ACB, or phosphate-buffered saline (PBS) for 1.5 h; each plate was agitated every 10 min. Then, DMEM containing 2% FBS was added to the cells that had been treated with rL-RVG, wild-type NDV, MLA, ACB or PBS, which was used as a negative control. After different periods of time, i.e., 24 h, 48 h or 72 h, 20 μL of MTT reagent (5 mg/mL) was added to each well. After 4 h of incubation, 100 μL of DMSO was added to each well, and the plates were agitated for 10 min. Finally, the absorbance was read at 490 nm using a standard spectrophotometer (FLX800, BioTek Instruments Inc., Shelburne, VT, USA). The above MTT experiment was repeated three times and the cell viability was calculated by the following formula. Cell viability = (the average A of the experimental group/blank control group A) * 100%.

#### Western blot analysis

Western blot was used to determine whether rL-RVG and NDV had infected the A549 cells. In addition, the expression levels of α7 nAChR, Bax, Bcl-2, caspase-3, caspase-8 and caspase-9 in A549 cells in each treatment group were examined after 48 h.

After the A549 cells were treated, 6-well plates were used with 80 μL/well of RIPA lysis buffer containing a protease inhibitor cocktail (Santa Cruz) to obtain protein samples. Then, the protein concentrations were measured using a BCA kit (Thermo Fisher Scientific, USA). Subsequently, each sample was combined with an equivalent volume of 2× loading buffer, subjected to 8–15% gradient SDS-PAGE, transferred to a PVDF membrane, and blocked for 1.5 h with 5% bovine serum albumin (BSA). Finally, the membranes were incubated with the following unique primary antibodies overnight at 4 °C: rabbit polyclonal anti-β-actin (1:5000); mouse monoclonal anti-RVG (1:200); chicken monoclonal anti-NDV (1:300); rabbit polyclonal anti-α7 nAChR (1:300); rabbit polyclonal anti-caspase-3 (1:300), anti-caspase-8 (1:300), and anti-caspase-9 (1:300); mouse monoclonal anti-Bcl-2 (1:200); and rabbit polyclonal anti-Bax (1:300). An incubation with corresponding HRP-conjugated secondary IgG antibodies (1:10,000) followed this step. The protein bands were detected by an image analyzer using the Luminata™ Crescendo Western HRP Substrate (Millipore, Middlesex, MA, USA).

#### Immunofluorescence analysis

The immunofluorescence analysis aimed to detect the expression of α7 nAChR, NDV and RVG protein in infected A549 cells in the different groups. A549 cells were seeded in 24-well plates at a density of 1 × 10^4^ cells per well. The next day, the cells were administered the following different treatments: rL-RVG or wild-type NDV at a multiplicity of infection (MOI) of 10 [11], MLA (10^−3^ mol/L), ACB (10^−3^ mol/L), MLA + RVG, ACB + RVG, or PBS as a negative control. At 24 h post-infection, the cells were fixed in ice-cold 4% paraformaldehyde (Solar Biotech Inc., Beijing, China) overnight at 4 °C; then, the cells were washed three times for 30 min with ice-cold PBS. After the cells were blocked with 3% BSA at 4 °C for 1 h, they were incubated with rabbit anti-α7 nAChR antibody (1:300), chicken anti-NDV antibody (1:300) and mouse anti-RVG antibody (1:200) overnight at 4 °C and were then washed three times for 30 min each with PBS. Next, the cells were stained with Cy3-conjugated goat anti-rabbit secondary antibody (1:300), Cy3-conjugated goat anti-chicken secondary antibody (1:300) or FITC-conjugated goat anti-mouse secondary antibody (1:100) for 2 h at room temperature and were washed three times for 1 h with PBS. The nuclei were stained with Hoechst 33,342 (2 μg/mL, Sigma-Aldrich) for 30 min, and then the cells were analyzed using a confocal laser microscope (Axio Observer A1, Zeiss, Germany).

#### TUNEL assay

A549 cells in the logarithmic growth phase were seeded on cover slips placed on the bottom of 24-well plates, incubated overnight, and then treated with the diluted rL-RVG or wild-type NDV at an MOI of 10 [11], MLA (10^−3^ mol/L), ACB (10^−3^ mol/L), MLA + RVG, ACB + RVG or PBS as negative control. TUNEL assay kits were used according to the manufacturer’s instructions to analyze apoptosis. At 48 h post-infection, the cells were fixed in ice-cold 4% paraformaldehyde (Solar Biotech Inc., Beijing, China) for 30 min at room temperature (15–25 °C); On the other hand, 5 μm paraffin sections of lung adenocarcinoma tissue were prepared at 60 °C for 60 min, rinsed in xylene for 10 min and immersed into the gradient ethanol. Then, the cells were washed for 30 min with PBS and incubated with 3% hydrogen peroxide in methanol for 10 min at room temperature. After being rinsed with PBS for 25 min, the samples were incubated with 0.1% Triton X-100 and 0.1% sodium citrate in water for 30 min at room temperature. The negative control samples did not receive TdT, and the positive control samples were treated with DNase I. After being washed with PBS for two hours, the pretreated specimens were incubated with 50 μL of TdT labeling reaction buffer at 4 °C overnight in the dark and then in a humidified atmosphere at 37 °C for an additional 2–3 h. Subsequently, the slides were incubated with 50 μL of streptavidin-HRP for 60 min, followed by detection with 50 μL of diaminobenzidine reagent for 10 min. The cells were observed and imaged under an optical microscope (ECLIPSE TS100, Nikon, Japan). This process was repeated three times. The apoptotic index (AI) was calculated for each sample by the following formula: the number of apoptotic cells/(the number of apoptotic cells + the number of non-apoptotic cells)×100%.

#### Immunohistochemistry

Sections of lung cancer and pericancerous tissues were fixed with 10% formaldehyde solution at 60 °C overnight. The paraffin sections were rinsed in xylene for 10 min twice and were dewaxed with an ethanol gradient (100, 95, 90, 80 and 70%) prior to undergoing antigen retrieval in boiling sodium citrate-hydrochloric acid buffer solution for 20 min. The sections were then washed with PBS three times, incubated with anti-α7 nAChR antibody (dilution ratio 1:100) at 4 °C overnight, washed with PBS for 5 min three times and incubated with anti-rabbit antibody (GT Vision™ I type polymer) (1:200) for 10 min. Then, the samples were washed with PBS for 5 min three times. All sections were incubated with HRP-conjugated secondary IgG antibodies (1:10,000) for 30 min at 37 °C, incubated with 3,3′-diaminobenzidine working solution for 10 min, washed with PBS for 5 min three times, and were then stained for 5 s with hematoxylin. Subsequently, the sections were washed with PBS for 5 min three times, dehydrated with an ethanol gradient (70, 80, 90, 95 and 100%) and rinsed in xylene for 10 min twice. Finally, the sections were observed under an optical microscope. Positive cells were indicated by claybank particles present in the cell membrane.

#### Statistical analysis

All data and results were analyzed using SPSS 16.0 software (International Business Machines, Armonk, NY, USA) and are expressed as the mean ± standard deviation. Statistically significant differences among the groups were analyzed using Student’s t-test for the MTT assay data and Western blot absorbance ratios and using one-way analysis of variance for the TUNEL assay data. *P* values <0.05 were considered to represent significant differences.

## Results

### Screening cell lines for the highest α7 nAChR expression

**Fig. 1:**
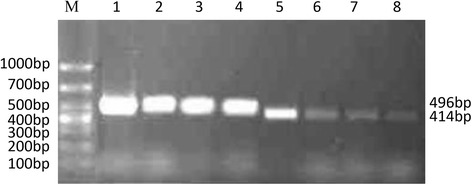
Gene expression of α7 nAChR in different tumor cell lines. (M) Marker. (1–4) GAPDH. A549, SCLC, U251 and LA795, respectively; (5–8) α7 nAChR. A549, SCLC, U251and LA795, respectively

RT-PCR was performed to identify the cell line with the high α7 nAChR expression. The result showed that the expression of the α7 nAChR gene (~414 bp) in A549 cells was higher than that in other cells. There was nearly no α7 nAChR gene expression in LΑ795 cells. Despite being derived from tumors of the nervous system, U251 cells did not have the highest level of α7 nAChR expression. On the other hand, α7 nAChR expression in SCLC cells was almost equivalent to that in U251 cells (Fig. 1).

### Screening for optimal agonist and antagonist concentrations

Our colleagues have previously screened for the optimal rL-RVG and NDV treatment concentration and duration; A549 cell viability and morphology were affected by treatment with rL-RVG or NDV for 48 h [7]. Thus, in this work, we only needed to determine the optimal treatment concentrations and durations for the receptor agonist and antagonist. The MTT results showed that the viability of A549 cells decreased with increasing antagonist concentration and incubation time following infection. However, the viability of agonist-treated A549 cells exhibited an opposite trend. The antagonist-treated cells also showed morphological changes. In contrast, no changes were observed in the PBS-treated and agonist-treated groups.

**Fig. 2:**
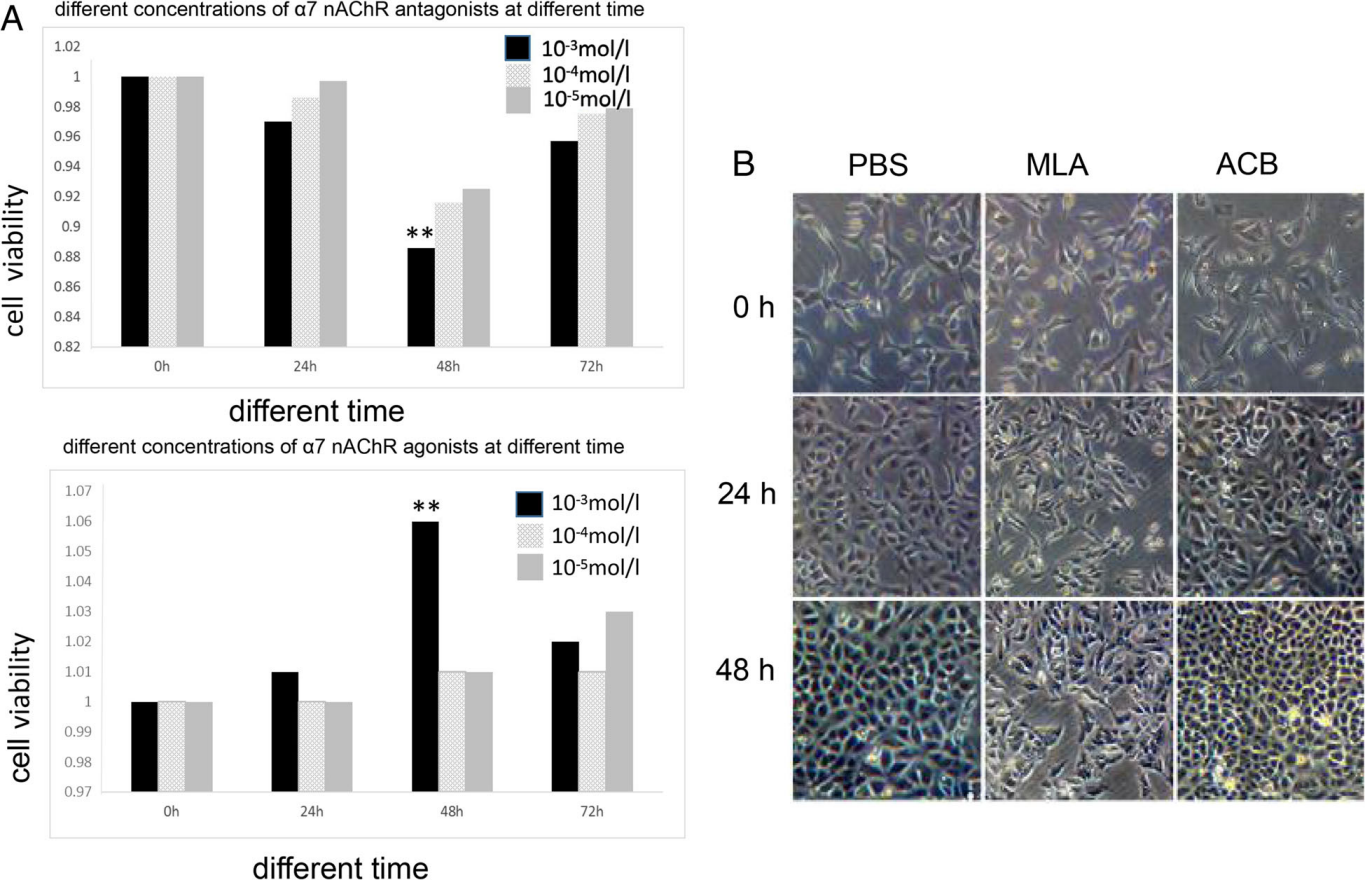
Changes of viability and morphology in antagonist-treated and agonist-treated cells. **a** Changes of viability in antagonist-treated and agonist-treated cells. **b** Changes of morphology in antagonist-treated and agonist-treated cells

The effect of different concentrations of agonist and antagonist on the logarithmic growth phase of A549 cells after 24 h and 48 h was evaluated by MTT assays. The results showed that the inhibition rates in the antagonist group were significantly greater than those in the PBS group, and the inhibition level increased with time following infection. However, the agonist significantly promoted cell proliferation, and the promotion level increased with time following infection (Fig. 2a). The optimal treatment durations and concentrations for both the antagonist and agonist in A549 cells were determined to be 48 h and 10^−3^ mol/L, respectively (Additional file 1: Tables S2-S3). The antagonist-treated and agonist-treated cells were also observed for changes in viability and morphology under the microscope.Antagonist-treated cells exhibited decreasing viability as well as morphological changes. However, agonist-treated cells exhibited increasing viability and no morphological changes compared with the PBS-treated cells (Fig. 2b).

### Expression of NDV and RVG genes and proteins in A549 cells after infection with rL-RVG and NDV

**Fig. 3:**
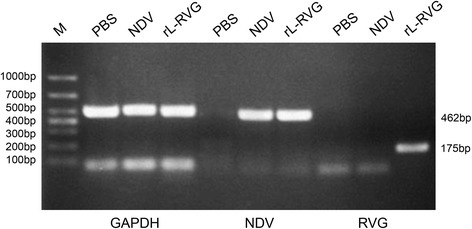
Expression of NDV and RVG genes in A549 cells after infection with rL-RVG and NDV. (M) Marker

A previous study of ours showed that the RVG was stably expressed in A549 cells by PCR, western blot and immunofluorescence analysis [7].In the present study, We only used RT-PCR to assess RVG and NDV gene expression in A549 cells following infection with rL-RVG and NDV. The results showed that the RVG gene (~175 bp) and NDV hemagglutinin-neuraminidase (HN) gene (~462 bp) were both expressed in rL-RVG-treated A549 cells. However, only the NDV HN gene (~462 bp) was expressed in NDV-treated A549 cells, and neither gene was expressed in the PBS-treated cells (Fig. 3a).

### Effects of rL-RVG, agonist and antagonist treatment on α7 nAChR gene and protein expression in A549 cells

**Fig. 4:**
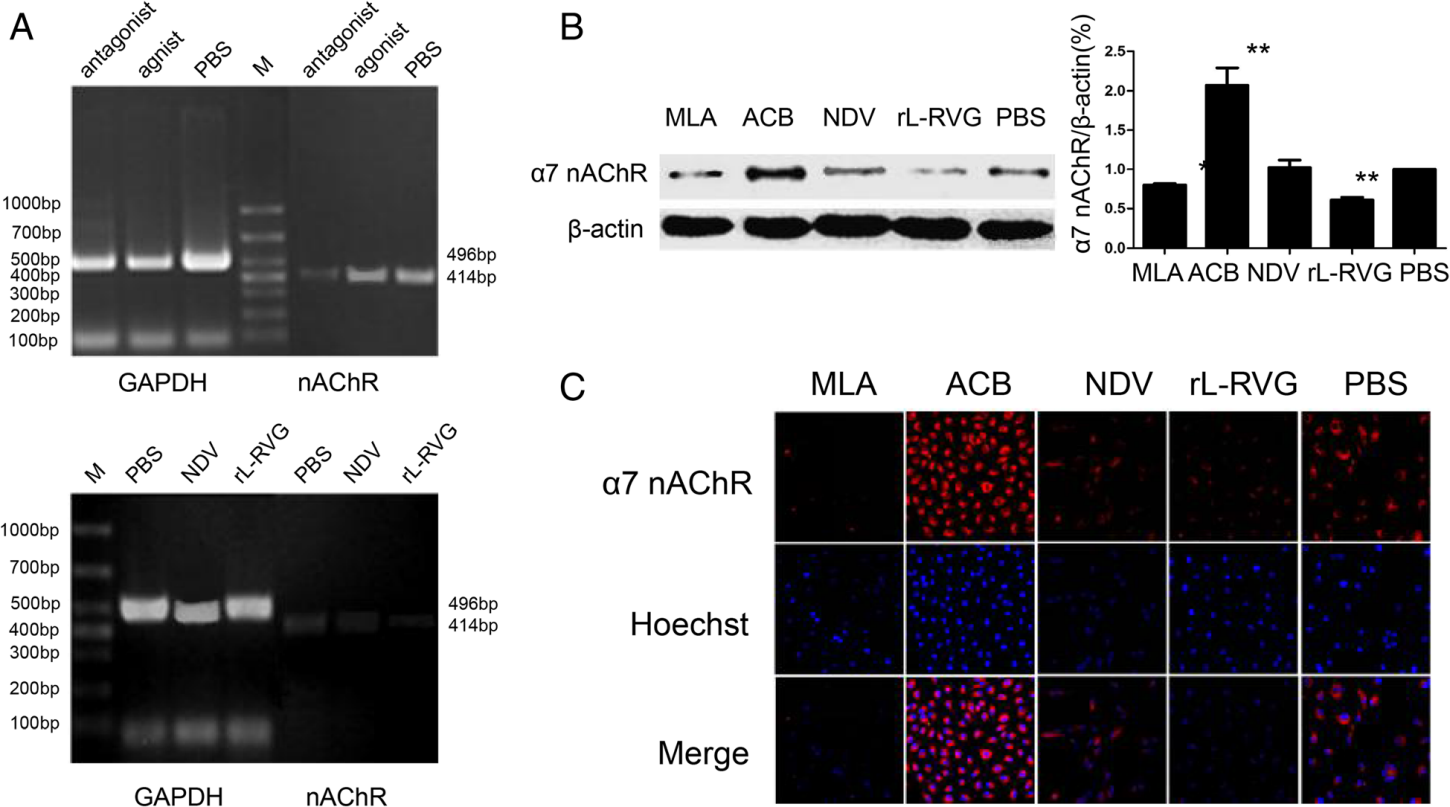
Effects of rL-RVG, agonist and antagonist treatment on α7 nAChR gene and protein expression in A549 cells. **a** Effects of the agonist, antagonist, rL-RVG and NDV on α7 nAChR gene expression in A549 cells. (M) Marker. **b** and **c**, α7 nAChR protein expression in A549 cells infected with rL-RVG and NDV

RT-PCR was performed to assess α7 nAChR gene expression in A549 cells following infection with rL-RVG and NDV and to determine the effects of the nAChR agonist and antagonist treatments. α7 nAChR gene expression was lower in the rL-RVG group than the NDV group. At the same time, α7 nAChR gene expression in the antagonist group was lower than that in the agonist and PBS groups (Fig. 4a).

Both Western Blot and immunofluorescence analyses were performed to assess α7 nAChR protein expression. The results showed that the rL-RVG and MLA treatments were negatively associated with α7 nAChR protein expression to different extents; the rL-RVG group exhibited weaker expression compared with the MLA group. However, the ACB treatment was positively associated with α7 nAChR protein expression, showing stronger expression than that in the PBS group. These results revealed that rL-RVG and MLA play the same role in inhibiting the expression α7 nAChR (Fig. 4b-c).

### Effects of rL-RVG, agonist and antagonist treatment on A549 cell apoptosis and migration

#### Changes in apoptosis-related protein expression

**Fig. 5:**
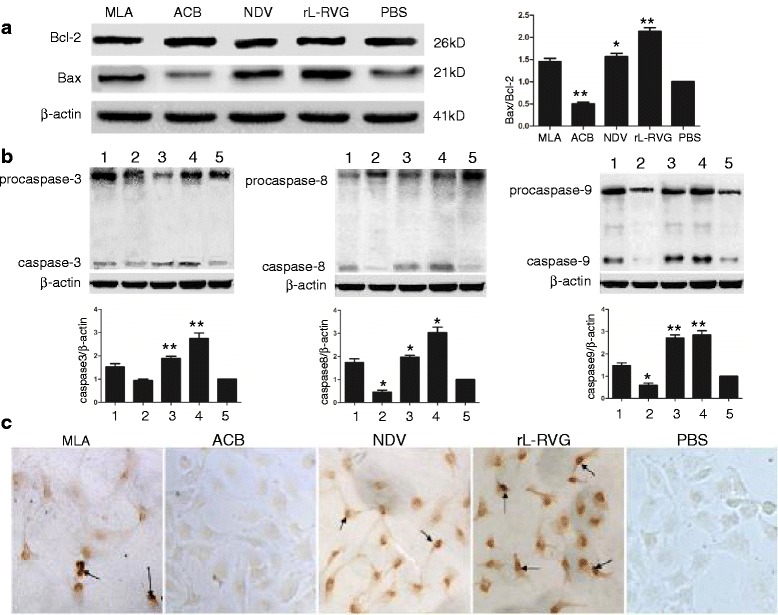
Effects of rL-RVG, agonist and antagonist treatment on apoptosis and related protein expression in A549 cells. **a** and **b** Apoptosis-related protein expression in A549 cells treated with rL-RVG, agonist and antagonist.(M) Marker;(1) MLA;(2) ACB;(3) NDV;(4) rL-RVG;(5) PBS. **c** Apoptosis in A549 cells treated with rL-RVG, agonist and antagonist (magnification,200×). The black arrows indicate TUNEL-positive nuclei

The expression levels of apoptosis-related proteins were examined by Western Blot; caspase-3, caspase-8, and caspase-9 activation and Bcl-2/Bax levels in A549 cells were assessed following infection with rL-RVG or NDV and treatment with MLA and ACB. Compared with the PBS group, a dramatic increase in caspase-3, caspase-8, caspase-9 and Bcl-2/Bax protein levels was observed in A549 cells after treatment with the antagonist (10^−3^ mol/L) for 48 h. In contrast, pretreating the A549 cells with the agonist (10^−3^ mol/L) for 48 h did not significantly reduce the apoptosis-related protein levels. The caspase-3, caspase-8, caspase-9 and Bcl-2/Bax levels were markedly higher in the rL-RVG and NDV groups than in the PBS group (*P*< 0.01), with the rL-RVG group exhibiting higher levels than the NDV group (*P*< 0.05). These protein levels were the highest in the rL-RVG group compared with the NDV, MLA, and ACB groups, and there was a significant difference between the rL-RVG and MLA groups (*P*< 0.05) (Fig. 5a-b).

#### TUNEL assay

The TUNEL assay was used to detect the occurrence of apoptosis following A549 cell treatment with rL-RVG, NDV, MLA, or ACB. TUNEL-positive nuclei were observed in the rL-RVG, NDV, and MLA groups; compared with these three groups, there was nearly no apoptosis observed in the ACB and PBS groups. The number of apoptotic cells and the AI were markedly higher in the rL-RVG and NDV groups than in the PBS group (*P*< 0.01), with the rL-RVG group exhibiting a higher number and AI compared with the NDV group (*P*< 0.05). The AI was the highest in the rL-RVG group compared with the NDV and MLA groups, and the AI in the NDV group was higher than that in the MLA group. In addition, there was a significant difference between the rL-RVG and MLA groups (*P*< 0.05) and the rL-RVG and NDV groups (*P*< 0.05) (Fig. 5c, Additional file 1: Table S4) after the A549 cells were treated with the agonist or antagonist for 48 h.

#### Effects of rL-RVG, agonist and antagonist treatment on A549 cell migration

**Fig. 6:**
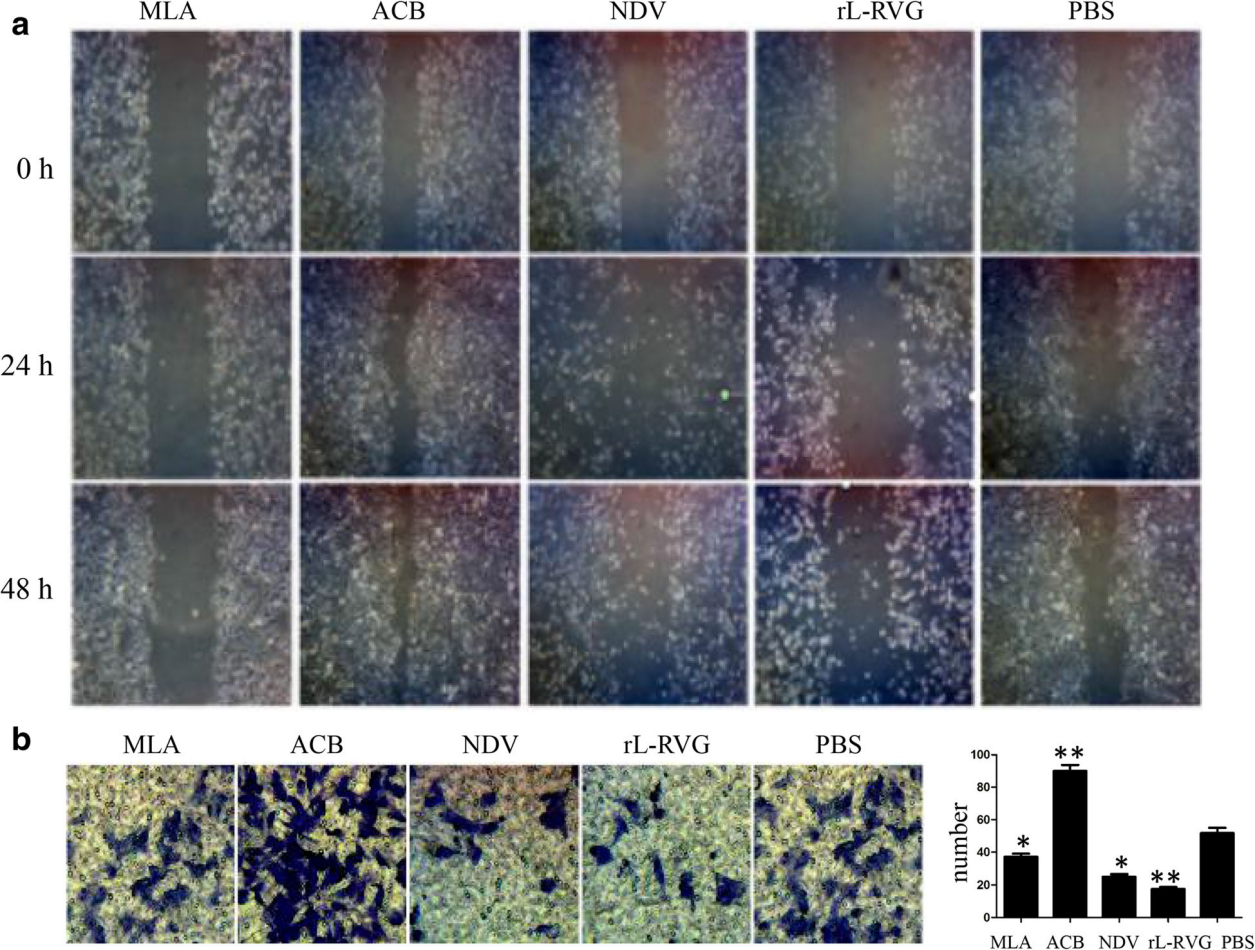
The migration of tumor cells after being treated with rL-RVG, agonist and antagonist. **a** Scratch test; (**b**) Transwell test

Scratch and transwell tests were used to investigate changes in the migration and invasion of A549 cells after treatment with the agonist and antagonist for 48 h. The transwell migration assay results suggested that the agonist-treated cells were more aggressive than the PBS-treated cells, but the antagonist significantly inhibited A549 cell invasion (*P*< 0.05). The scratch test demonstrated that the agonist positively affected migration capacity while the antagonist had a significant negative effect on cell migration. On the other hand, the results of scratch and transwell tests both showed that the rL-RVG and NDV treatments inhibited tumor cell migration, and the rL-RVG treatment inhibited A549 cell migration more than did the NDV (*P*< 0.05), agonist and antagonist treatments (Fig. 6a-b).

### rL-RVG + ACB and NDV + MLA affect A549 cell apoptosis and migration ability

#### The level of A549 cell apoptosis

**Fig. 7:**
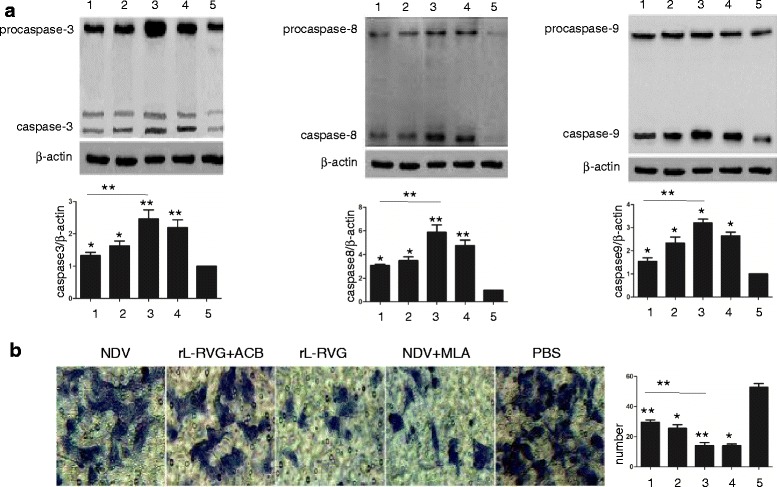
A549 cell apoptosis-related protein levels and migration after the various treatments. **a** Apoptosis-related protein levels in the treated A549 cells. (1) NDV group; (2) rL-RVG + ACB group; (3) rL-RVG group; (4) NDV + MLA group; (5) PBS group. **b** The migration of treated A549 cells as determined by a transwell test

To examine whether the greater suppression of lung cancer cell invasion exerted by the rL-RVG strain compared with the NDV strain was related to altered α7 nAChR expression, we tested three different treatments. A549 cells were first pretreated with ACB or MLA and were then infected with rL-RVG or NDV; rL-RVG, NDV and PBS treatments alone were used as controls. The Western blot analysis results showed that all four groups exhibited greater A549 cell apoptosis compared with the PBS group. The expression levels of the apoptosis-related proteins caspase-3, caspase-8, and caspase-9 were higher in the NDV + MLA and rL-RVG groups than in the NDV and rL-RVG + ACB groups. In addition, no significant differences were found between the NDV + MLA and rL-RVG groups and the rL-RVG + ACB and NDV groups (Fig. 7a).

#### Tumor cell migration ability after treatment with rL-RVG + ACB or NDV + MLA

The transwell migration assay was used to investigate the effect of rL-RVG + ACB and NDV + MLA treatment on A549 tumor cell migration ability after 48 h. The results suggested that the cells treated with rL-RVG + ACB were more aggressive than those treated with rL-RVG alone. In addition, the NDV + MLA treatment inhibited tumor cell invasion compared with the NDV treatment (*P*< 0.05), but there was no significant difference between the rL-RVG + ACB and NDV + MLA groups (*P*> 0.05) (Fig. 7b).

#### The level of α7 nAChR expression and apoptosis in lung cancer tissue and its relationship with clinical characteristics

**Fig. 8:**
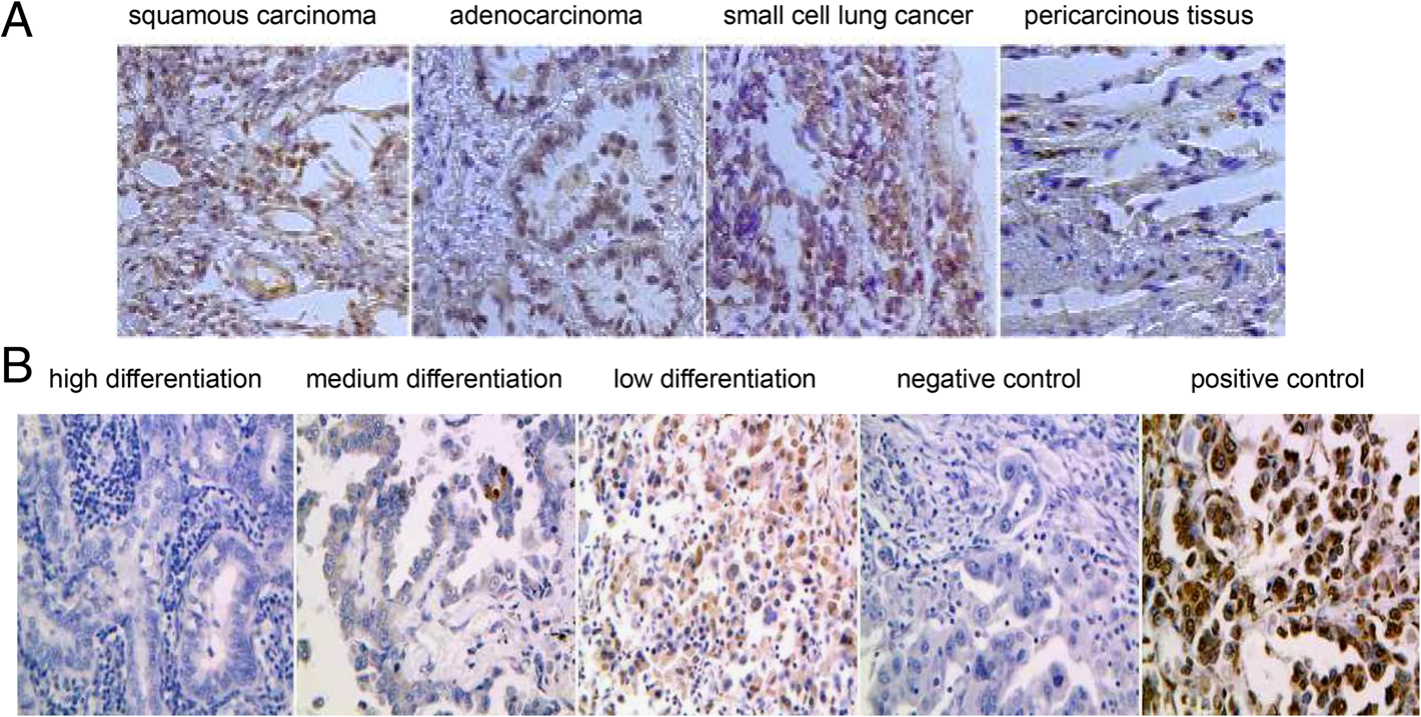
The expression of α7 nAChR and apoptosis in lung cancer. **a** The expression of α7 nAChR in various lung cancer tissues, including squamous carcinoma, adenocarcinoma and SCLC, as well as in pericancerous tissues. **b** The apoptosis in lung adenocarcinoma tissues in low, medium and high differentiation groups

The expression of α7 nAChR in lung cancer tissue and pericancerous tissues α7 AChR-positive staining was found in various lung cancer tissues, including squamous carcinoma, adenocarcinoma and SCLC, as well as in pericancerous tissues; the staining was localized in the cell membrane. However, the level of α7 nAChR expression in the pericancerous tissues was significantly lower than that in the cancer tissues, with 37.50% and 70.77% positive staining for α7 nAChR, respectively (*P*< 0.001) (Fig. 8a).

The level of α7 nAChR expression in lung cancer tissue isolated from lung cancer patients was correlated with smoking, clinical stage and differentiation degree (*P*< 0.05). There were no significant differences in the level of α7 nAChR expression in lung cancer tissue according to gender, age, tumor size, pathological type or lymph node metastasis (*P*> 0.05) (Additional file 1: Table S5). The apoptosis index (AI) in lung adenocarcinoma tissue in high-medium differentiation group and low differentiation group was 2.587 ± 0.1878,3. 597 ± 0.1472, respectively (*p* < 0.01) (Fig. 8b).

## Discussion

NDV is classified as an avian paramyxovirus-1 in the *Avulavirus* genus of the family *Paramyxoviridae* [13], whose genomes consist of negative-sense single-stranded RNA. This RNA contains six open reading frames that encode six structural proteins: the nucleoprotein, the phosphoprotein, the large polymerase protein, the matrix protein, HN and fusion glycoproteins [14]. The infection of host cells starts once the viral HN protein binds to its receptor [15]. Receptor recognition by HN triggers the activation of the fusion protein, which promotes the fusion of the viral and cell membranes and allows the entry of the ribonucleoproteins into the cytoplasm. Genome replication takes place in the cytoplasm and does not involve any intermediate DNA stages.

The rL-RVG strain was provided by the Harbin Veterinary Research Institute (Harbin, China); it is a recombinant NDV expressing RVG. Recent research has demonstrated that rL-RVG can spread between cells in a similar manner as the rabies virus does. However, RVG does not alter the infection ability of NDV in mammalian cells [8]. NDV was considered to be a promising viral oncolytic therapeutic because it could stimulate host cell apoptosis [16]. In addition, oncolytic NDV has been investigated for the suppression of proliferation and invasion in glioblastoma multiforme [17]. NDV induced caspase-dependent apoptosis, and caspase-3, caspase-8, and caspase-9 are known to play different roles in the activation of the extrinsic and intrinsic pathways [18]. On the other hand, Hu L et al. found that NDV LaSota triggered apoptosis in A549 human lung adenocarcinoma cells through upregulating the anti-apoptosis protein Bax [19]. Consistent with these discoveries, our previous study showed that rL-RVG was able to suppress lung cancer cell growth and promote lung cancer cell apoptosis to a greater extent than the wild-type NDV strain, as well as dramatically increase the levels of the apoptosis-related proteins caspase-3, caspase-8, caspase-9 and Bax in A549 cells [7].

Nevertheless, the underlying mechanism of rL-RVG-induced anti-tumor activity has not yet been revealed. RVG and AChR antagonists have a high degree of homology; the amino acid sequence of RVG from 198 to 214 is highly homologous with that of α–bungarotoxin, which is an α7 nAChR-specific antagonist. In addition, purified RVG was demonstrated to be able to compete with the potent neurotoxin of the snake *Bungarus multicinctus*, a-bungarotoxin, for acetylcholine receptor binding [20, 21]. nAChRs have been known to be crucial regulators of cancer cells since the early 1980s, and the homomeric α7 nAChR accelerates proliferation, migration, angiogenesis, neurogenesis and metastasis in most common human cancers by stimulating the synthesis and release of excitatory neurotransmitters while inhibiting apoptosis [22]. Nicotine, an α7 nAChR agonist, can facilitate tumor development and progression [23, 24]. Some studies show that nicotine significantly augments the progression and metastasis of tumors in mouse models of lung cancer [25]. α7 nAChR plays an essential role in acute lung injury induced by oxidative stress and inflammation through TLR4/NF-κB signaling suppression, and an MLA treatment aggravated the apoptosis in the lung [26]. As α7 nAChR ligands can be positive allosteric modulators, there is renewed hope for treatment discovery [27]. We speculate that rL-RVG, as an α7 nAChR antagonist, can promote lung cancer cell apoptosis and suppress its invasion capability. Thus, this study focused on the relationship between α7 nAChR expression and rL-RVG-induced apoptosis, A549 cell invasion and the clinical characteristics of lung cancer.

The RT-PCR results showed that the α7 nAChR gene (~414 bp) was expressed in the A549, LΑ795, SCLC and U251 cell lines and that the highest expression was in A549 cells; as such, these cells were selected for use in subsequent studies. The RT-PCR results showed that rL-RVG and NDV could both effectively infect A549 cells. We chose MLA as a specific antagonist and ACB as an agonist of α7 nAChR in A549 cells. The MTT assay results showed that the optimal treatment duration and concentration for both the agonist and antagonist in A549 cells was 48 h and 10^−3^ mol/L, respectively.

Subsequent PCR, Western blot and immunofluorescence results indicated that the rL-RVG and MLA treatments were negatively associated with α7 nAChR gene and protein expression to different extents and that the rL-RVG group exhibited weaker α7 nAChR expression compared with the MLA group. On the other hand, the α7 nAChR agonist promoted migration and inhibited apoptosis in A549 lung cancer cells, while the antagonist had opposite effects. Both the rL-RVG and NDV treatments inhibited the migration and increased the apoptosis of the tumor cells. In addition, the rL-RVG treatment inhibited of A549 cell migration more than did the NDV, agonist and antagonist treatments, and there was a significant difference between the rL-RVG and MLA groups. These results were as expected, so we explored further. To confirm whether rL-RVG inhibiting the α7 nAChR pathway contributed to the induction of apoptosis and the inhibition of migration, we distributed A549 cells into five groups, i.e., the NDV, rL-RVG + ACB, rL-RVG, NDV + MLA and PBS groups. The results revealed that there was no significant difference in A549 migration and apoptosis between the NDV and rL-RVG + ACB groups and the rL-RVG and NDV + MLA groups; the rL-RVG and NDV + MLA groups had higher rates of invasion inhibition and apoptosis promotion in A549 cells. These results supported the hypothesis that rL-RVG partially enhanced the apoptosis and inhibited the migration of A549 lung adenocarcinoma cells by downregulating α7 nAChRs.

α7 nAChRs can stimulate proliferation in a variety of normal and tumor cells, such as small-cell carcinoma, NSCLC, pancreatic cancer, breast cancer, bladder cancer and colon cancer cells [28]. α7 nAChRs may activate β2-adrenergic dependent SRC, epidermal growth factor receptor, AKT, Survivin, NF-κB and the epithelial-mesenchymal transition to promote the proliferation of NSCLC cells through the RAS system [29–33] and promote the proliferation, angiogenesis, invasion and metastasis of lung cancer cells [22, 34–36]. Zovko et al. found that APS8 can block the activity of α7 nAChR, leading to the apoptosis of NSCLC cells [37].

After analyzing α7 nAChR expression levels and the related clinical characteristics, the results showed that the intensity and rate of positive α7 nAChR expression in lung cancer tissues were significantly higher than those in pericancerous tissues, suggesting that α7 nAChRs might be involved in the occurrence and development of lung cancer. The results further confirmed that the expression intensity of α7 nAChRs increased with increasing clinical stage and decreasing differentiation degree; on the other hand, the apoptosis in lung adenocarcinoma tissue also augmented with decreasing differentiation degree. These above finding suggests that α7 nAChR expression might be an important precipitating factor of lung cancer and that lung cancer patients with positive α7 nAChR activity might have a poor prognosis.

Nicotine promotes the growth and metastasis of tumors mainly by activating α7 nAChRs [38], and α7 nAChR antagonists can inhibit nicotine-mediated angiogenesis in lung tumors [39]. A variety of downstream signaling cascades can be triggered by nicotine through α7 nicotinic receptors, including the Ras/Raf-1/MEK1/ERK and JAK-2/STAT-3 pathways, resulting in the progression of cancer [40, 41]. A recent study demonstrated that nicotine and α7 nAChRs enhance lung cancer promotion via the HGF-induced PI3K/Akt signaling pathway [42].

As α7 nAChRs may play an important role in the pathological process of lung cancer growth, invasion, and transformation, detecting the level of α7 nAChR expression for the early diagnosis of lung cancer and determining patient prognosis as part of a comprehensive evaluation has a certain guiding significance. Taken together, these results suggest that rL-RVG enhances the apoptosis and inhibits the migration of A549 lung adenocarcinoma cells by regulating α7 nAChR signaling and eliminating tumor cells. An α7 nAChR antagonist could be used as a molecular target for lung adenocarcinoma therapy. However, the mechanisms of the rL-RVG-induced anti-tumor effects exerted via α7 nAChR and downstream signaling regulation remain unclear. Further research is required to determine the efficacy and safety of potential rL-RVG and MLA tumor treatments. Future studies are also required to explore the effects of nicotinic acetylcholine receptor antibodies and siRNA on the proliferation, development, and invasion of lung cancer cells, so as to target α7 nAChR in treating lung cancer.

## Conclusions

α7 nAChRs might be related to the occurrence and development of lung adenocarcinoma. Recombinant NDV rL-RVG significantly not only inhibited proliferation and migration, but also promoted apoptosis in lung adenocarcinoma cells by regulating α7 nAChR signaling pathways. An antagonist of α7 nAChR may be a molecular target for lung adenocarcinoma treatment.

## Additional file

Additional file 1:
**Tables S1.** Primers used for PCR amplification. **Table S2.** The impact of proliferation after A549 cells were treated with different concentrations of an α7 nAChR agonist for different times $\left(\overline{x}\pm \mathrm{s}\right)$. **Table S3.** The impact of proliferation after A549 cells were treated with different concentrations of an α7 nAChR antagonist for different times $\left(\overline{x}\pm \mathrm{s}\right)$. **Table S4.** The AI in A549 cells treated with rL-RVG, agonist and antagonist. **Table S5.** Relationship between α7 nAChR expression and clinical factors. (DOC 91 kb)

## Abbreviations

BSA: Bovine serum albumin
MOI: Multiplicity of infection
MTT: 3-[4, 5-Dimethyl-2-thiazolyl]-2, 5-diphenyl-2H-tetrazolium bromide
NAChRs: Neuronal nicotinic acetylcholine receptors
NDV: Newcastle disease virus
NSCLC: Non-small-cell lung cancer
PBS: Phosphate-buffered saline
rL-RVG: Recombinant Newcastle disease virus
RT-PCR: Reverse transcription polymerase chain reaction
SCLC: Small-cell lung carcinoma
α7 nAChRs: Alpha 7 nicotinic acetylcholine receptors
