# Supplementary material for: Recombinant Newcastle disease virus rL-RVG enhances the apoptosis and inhibits the migration of A549 lung adenocarcinoma cells via regulating alpha 7 nicotinic acetylcholine receptors in vitro
Source: Virol J. 2017 Oct 3;14:190. doi: 10.1186/s12985-017-0852-z (PMC5627431; doi:10.1186/s12985-017-0852-z)
Supplement: Additional file 1: — Tables S1. Primers used for PCR amplification. Table S2. The impact of proliferation after A549 cells were treated with different concentrations of an α7 nAChR agonist for different times $\left(\overline{x}\pm \mathrm{s}\right)$x¯±s. Table S3. The impact of proliferation after A549 cells were treated with different concentrations of an α7 nAChR antagonist for different times $\left(\overline{x}\pm \mathrm{s}\right)$x¯±s. Table S4. The AI in A549 cells treated with rL-RVG, agonist and antagonist. Table S5. Relationship between α7 nAChR expression and clinical factors. (DOC 91 kb) [file 12985_2017_852_MOESM1_ESM.doc]

**Tabel S1-S5**

**Table S1**. Primers used for PCR amplification.

| Primer | Sequence | Product size (bp) |
| --- | --- | --- |
| rL-RVG |  |  |
| Upstream | 5' AGCCGATGCTCACTACAAG 3' | 175 |
| Downstream | 5' CTGGAGGAGGGATGATTGC 3' |  |
| NDV |  |  |
| Upstream | 5' CTGGACGGTTTGGTGGGAA 3' | 462 |
| Downstream | 5' TAATGCGACTGCGGGATGTG 3' |  |
| α7 nAChR |  |  |
| Upstream | 5' CCTGGCCAGTGTGGAG 3' | 414 |
| Downstream | 5' TACGCAAAGTCTTTGGACAC 3' |  |
| GAPDH |  |  |
| Upstream | 5' CAAGGTCATCCATGACAACTTTG 3' | 496 |
| Downstream | 5' GTCCACCACCCTGTTGCTGTAG 3' |  |

rL-RVG, LaSota NDV strain expressing rabies virus glycoprotein; NDV, Newcastle disease virus; α7 nAChR, α7 nicotinic acetylcholine receptor.

**Table S2.** The impact of proliferation after A549 cells were treated with different concentrations of an α7 nAChR agonist for different times (
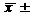
s).

| 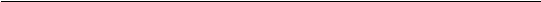group | *n* | 0 h | 24 h | 48 h | 72 h |
| --- | --- | --- | --- | --- | --- |
| control | 5 | 0.1815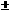0.0013 | 0.5094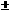0.0092 | 0.8519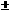0.0387 | 1.1401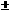0.0073 |
| 10^-3^mol/L | 5 | 0.1812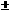0.0011 | 0.4983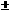0.0117 | 0.7549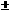0.0070^a b^ | 1.0921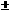0.0237 |
| 10^-4^mol/L | 5 | 0.1813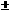0.0019 | 0.5010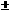0.0010 | 0.7800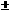0.0187^a^ | 1.1117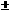0.0588 |
| 10^-5^mol/L | 5 | 0.1811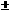0.0008 | 0.5080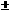0.0074 | 0.7881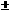0.0120^a^ | 1.1163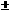0.0634 |
| *F* value |  | 0.0796 | 2.0282 | 15.7363 | 0.8227 |
| *P* value |  | 0.9702 | 0.1505 | 0.0000 | 0.5003 |

a: *P*＜0.01, compared with the control group; b: *P*＜0.05, compared with the 10^-4^ mol/L and 10^-5^ mol/L groups; 10^-3^ mol/L and 48 h were the optimal treatment concentration and duration in antagonist-treated A549 cells.

**Table S3.** The impact of proliferation after A549 cells were treated with different concentrations of an α7 nAChR antagonist for different times (
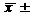
s).

| group | *n* | 0 h | 24 h | 48 h | 72 h |
| --- | --- | --- | --- | --- | --- |
| group | 5 | 0.1232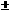0.0077 | 0.2999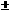0.0079 | 0.3651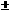0.0126 | 0.5930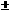0.0221 |
| 10^-3^ mol/L | 5 | 0.1240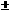0.0058 | 0.3042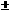0.0112 | 0.3879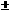0.0050^a^ | 0.6031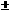0.094 |
| 10^-4^ mol/L | 5 | 0.1238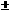0.0075 | 0.3017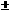0.0053 | 0.3699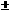0.0044 | 0.5999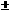0.0153 |
| 10^-5^ mol/L | 5 | 0.1333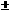0.0087 | 0.3009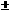0.0141 | 0.3689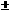0.0066 | 0.6079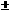0.0073 |
| *F* value |  | 2.0910 | 0.1635 | 8.4473 | 0.9036 |
| *P* value |  | 0.1417 | 0.9194 | 0.0014 | 0.4611 |

a: *P*＜0.01, compared with the control, 10^-4^ mol/L and 10^-5^ mol/L groups; the optimal treatment duration and concentration were 48 h and 10^-3^ mol/L in agonist-treated A549 cells.

**Table S4.** The AI in A549 cells treated with rL-RVG, agonist and antagonist.


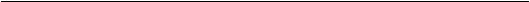
 group mean ± SD(n=10) F value P value


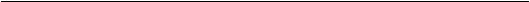


MLA 0.17415±0.02352 ^a^

ACB 0.04238±0.05029 ^b^

NDV 0.71635±0.03128 ^a^ 1.305E3 0.000

rL-RVG 0.90920±0.03878 ^a^

PBS 0.05195±0.02707


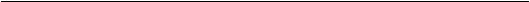
The AI was the highest in the rL-RVG group compared with the NDV and MLA groups, and the AI in the NDV group was higher than that in the MLA group. ^a^ P<0.01 versus the other groups. ^b^ P<0.01 versus the MLA, NDV and rL-RVG groups.

**Table S5.** Relationship between α7 nAChR expression and clinical factors

| Clinical features | cases | negative | positive | value | P value |
| --- | --- | --- | --- | --- | --- |
| Sex |  |  |  |  |  |
| Male | 74 | 22（29.73） | 52（70.27） | 0.021 | 0.886 |
| Female | 56 | 16（28.57） | 40（71.43） |  |  |
| Age |  |  |  |  |  |
| ＜60 | 33 | 10（30.30) | 23(69.70) | 0.025 | 0.875 |
| ≥60 | 97 | 28（28.87） | 69（71.13） |  |  |
| Smoking |  |  |  |  |  |
| Yes | 56 | 6（10.71) | 50(89.29) | 16.305 | ＜0.001 |
| No | 74 | 32(30.77) | 42(56.76) |  |  |
| Tumor size |  |  |  |  |  |
| ≤3 cm | 26 | 6（23.08） | 18（69.23） | 0.255 | 0.614 |
| ＞3 cm | 104 | 32（30.77） | 74（71.15） |  |  |
| Lymphatic metastasis |  |  |  |  |  |
| No | 25 | 5（20.00） | 20（80.00） | 1.474 | 0.225 |
| Yes | 105 | 34（32.38） | 71(67.62) |  |  |
| TNM stage |  |  |  |  |  |
| I-II | 78 | 8(10.26) | 70(89.74) | 33.938 | ＜0.001 |
| III-IV | 52 | 30(57.69) | 22(42.31) |  |  |
| Differentiation degree |  |  |  |  |  |
| High-medium | 88 | 15（17.05） | 73（82.95） | 19.551 | ＜0.001 |
| Low | 42 | 23（54.76） | 19（45.24) |  |  |
| Pathological type |  |  |  |  |  |
| Squamous carcinoma | 42 | 8（19.05) | 34(80.95) | 4.437 | 0.109 |
| Adenocarcinoma | 65 | 20(30.77) | 45(69.23) |  |  |
| Small-cell carcinoma | 23 | 10(43.48) | 13(56.52) |  |  |
